# Development of synaptic connectivity onto interneurons in stratum radiatum in the CA1 region of the rat hippocampus

**DOI:** 10.1186/1471-2202-13-14

**Published:** 2012-01-25

**Authors:** Ilse Riebe, Eric Hanse

**Affiliations:** 1 Department of Physiology, Sahlgrenska Academy, University of Gothenburg, Göteborg, Sweden

## Abstract

**Background:**

The impact of a given presynaptic neuron on the firing probability of the postsynaptic neuron critically depends on the number of functional release sites that connect the two neurons. One way of determining the average functional synaptic connectivity onto a postsynaptic neuron is to compare the amplitudes of action potential dependent spontaneous synaptic currents with the amplitude of the synaptic currents that are independent of action potentials ("minis"). With this method it has been found that average synaptic connectivity between glutamatergic CA3 and CA1 pyramidal cells increases from single connections in the neonatal rat, to multiple connections in the young adult rat. On the other hand, γ-aminobutyric acid (GABA)ergic interneurons form multiple connections onto CA1 pyramidal cells already in the neonatal rat, and the degree of multiple GABAergic connectivity is preserved into adulthood. In the present study, we have examined the development of glutamate and GABA connectivity onto GABAergic CA1 stratum radiatum interneurons in the hippocampal slice, and compared this to the connectivity onto CA1 pyramidal neurons.

**Results:**

In GABAergic interneurons in the CA1 stratum radiatum, irrespective of developmental stage, we found that the average amplitude of action potential dependent spontaneous AMPA receptor-mediated synaptic currents were of the same magnitude as the mini AMPA receptor mediated synaptic currents. This finding indicates that these GABAergic interneurons, in contrast to the CA1 pyramidal neurons, preserve single glutamate connectivity throughout development. For GABA connectivity, on the other hand, we found multiple functional synaptic connections onto the interneurons, as onto the pyramidal cells.

**Conclusions:**

The results presented here confirm that glutamate and GABA synaptic connectivity develop very differently in the hippocampal CA1 region. Thus, whereas average GABA connectivity is multiple throughout the development, glutamate connectivity is unitary early in development. Our results further suggest that the development of glutamate synaptic connectivity differs markedly between pyramidal cells and GABAergic interneurons in stratum radiatum, such that a given presynaptic glutamatergic cell appears not allowed to increase its connectivity onto the postsynaptic stratum radiatum interneuron, as it may do onto the postsynaptic CA1 pyramidal cell.

## Background

The number of functional release sites connecting two neurons critically contributes to decide the impact that the presynaptic neuron has on the firing probability of the postsynaptic neuron [1,2]. This synaptic connectivity varies from one, or zero for silent synapses, to many hundreds at specialized multi release site connections like the climbing fibre synapse in the cerebellum, the mossy fibre CA3 synapse in the hippocampus, or the Calyx of Held in the brainstem [3]. Most intracortical connections have, however, fewer than ten release sites [3]. What determines these differences in connectivity between different types of neuronal connections, and how the connection specific connectivity is established during brain development, is largely unknown.

In the rat hippocampal CA1 region the average glutamatergic synaptic connectivity between CA3 and CA1 pyramidal cells increases with development [4]. During the first ten postnatal days the connectivity is either one [4,5], or zero for the silent connections [4,6-9], whereas it thereafter increases to, on average, about five in the adult stage [4]. In striking contrast to the establishment of multiple glutamatergic connectivity onto CA1 pyramidal cells over a prolonged developmental period, multiple γ-aminobutyric acid (GABA)ergic connectivity onto CA1 pyramidal cells is established already during the first postnatal days [5]. Moreover, although there is an increasing number of GABAergic synapses, the average degree of GABAergic connectivity remains unaltered into adulthood [5]. These findings indicate that newly formed glutamatergic connections are restricted to one release site, whereas such restriction is lacking for newly formed GABAergic connections. These conclusions have been obtained by comparing the average amplitude of spontaneous action potential dependent postsynaptic currents (sPSCs), to that of action potential independent PSCs (miniature PSCs, mPSCs or "minis"). This amplitude ratio is referred to as multiplicity [4], and if each presynaptic cell forms at most one synaptic contact with the postsynaptic cell the multiplicity will be 1. If, on the other hand, each presynaptic cell forms more than one synaptic contact with the postsynaptic cell the multiplicity will be > 1, since the sPSCs will, on average, have a larger amplitude than the mPSCs. If the release probability is also taken into account it is possible to convert multiplicity to connectivity. Studies in the CA3 and CA1 region have shown good resemblance between electrophysiological and morphological estimates of connectivity [10-12].

Together these studies suggest that the mechanisms governing average connectivity between CA3-CA1 glutamatergic neurons and those governing average connectivity between GABAergic interneurons-CA1 glutamatergic neurons are fundamentally different, implying that the type of presynaptic neuron critically contributes to the connectivity properties. It is likely that the type of postsynaptic neuron is also important in deciding connectivity. Previous studies in the CA3 region, and of CA1 stratum oriens interneurons, have indicated that glutamatergic synaptic connectivity onto GABAergic interneurons may be lower than that onto pyramidal cells [12-14], whereas GABAergic connectivity onto GABAergic interneurons has not been examined. In the present study we have used the multiplicity approach to examine the development of glutamatergic and GABAergic connectivity onto stratum radiatum interneurons. Together with previous studies we conclude that GABAergic interneurons in stratum radiatum and pyramidal cells differ with respect to the development of glutamatergic connectivity, but exhibit similar development of GABAergic connectivity.

## Results

### Connectivity at CA3-CA1 pyramidal cell connections

**Figure 1:**
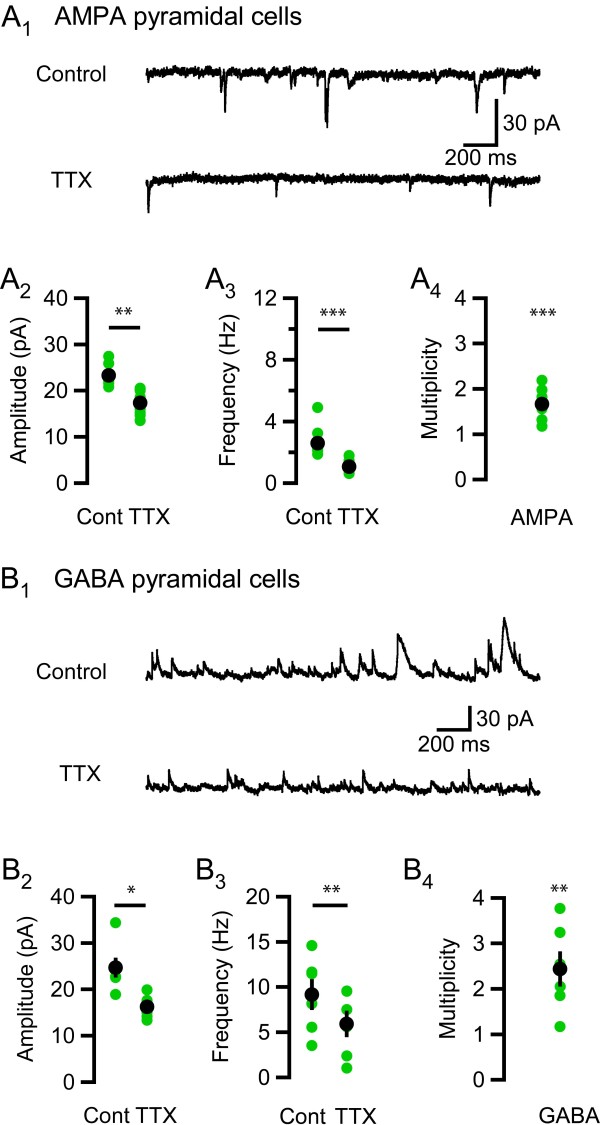
**Multiple connections between CA3 and CA1 pyramidal cells and GABAergic cells and CA1 pyramidal cells in young adult rats**. (A_1_) A typical recording at -80 mV from a pyramidal cell, upper trace shows AMPA sEPSCs under control conditions and lower trace shows mEPSCs after wash-in of TTX. (A_2_) TTX reduces the AMPA EPSC amplitude by 5.9 ± 0.8 pA (P = 0.004, n = 8, two-tailed paired t-test). (A_3_) TTX reduces AMPA EPSC frequency by 1.5 ± 0.4 Hz (P < 0.001, n = 8, one-tailed paired t-test). (A_4_) The average multiplicity was 1.7 ± 0.1 (P < 0.001, n = 8, one-tailed one-sample t-test with test value = 1). (B_1_) A typical recording at 0 mV from a pyramidal cell, upper trace shows GABA sIPSCs under control conditions and lower trace shows mIPSCs after wash-in of TTX. (B_2_) TTX reduces the GABA IPSC amplitude by 8.4 ± 2.7 pA (P = 0.03, n = 6, two-tailed paired t-test). (B_3_) TTX reduces GABA IPSC frequency by 3.2 ± 0.8 Hz (P = 0.005, n = 6, one-tailed paired t-test). (B_4_) The average multiplicity was 2.4 ± 0.4 (P = 0.007, n = 6, one-tailed one-sample t-test with test value = 1). Black filled circles show mean ± SEM and green filled circles show data from individual experiments. Asterisks denote the level of significance as follows: *: P < 0.05, **: P < 0.01, ***: P < 0.001.

Previous studies have shown that the average magnitude of spontaneous AMPA EPSCs in the presence of TTX (mEPSCs) and in the absence of TTX (sEPSCs) is the same in developing (< P10) rat CA1 pyramidal cells [4,15]. In contrast, in mature CA1 pyramidal cells the magnitude of action potential dependent AMPA sEPSCs is about two times larger than that of the AMPA mEPSCs [4]. We first wanted to confirm these previous findings. Figure 1A1 shows a typical recording from one pyramidal cell from a young adult (P30-60) rat before and after application of TTX (0.5 μM). Consistent with the findings of Hsia et al [4], the reduction in frequency induced by TTX (Figure 1A2, average reduction of 1.5 ± 0.4 Hz, P < 0.001, one-tailed paired t-test, only experiments with a reduction in frequency were included, see Methods for detailed description) was associated with a reduction in the amplitude of AMPA EPSCs (Figure 1A3, 5.9 ± 0.8 pA, P = 0.004, two-tailed paired t-test). Multiplicity calculations (see Methods) showed that the amplitude of action potential dependent AMPA sEPSCs was on average 1.7 ± 0.1 times larger than the AMPA mEPSC amplitude (P < 0.001, one-tailed one-sample t-test with test value = 1, n = 8, Figure 1A4).

### Connectivity at GABAergic interneuron - pyramidal cell connections

**Figure 2:**
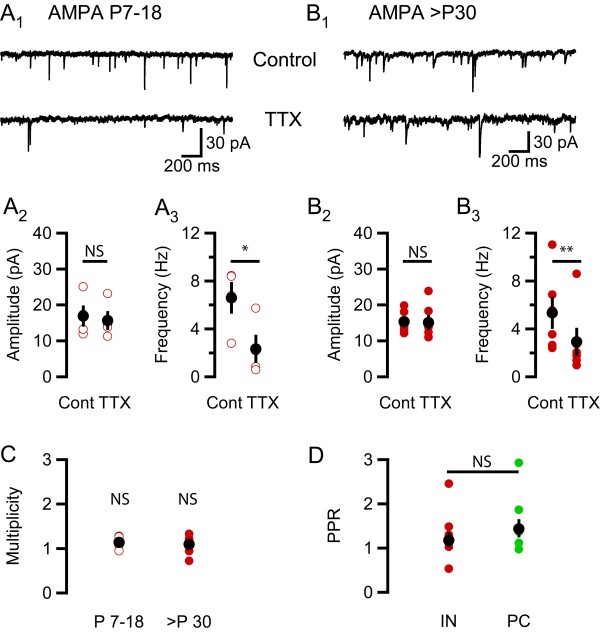
**No developmental change in multiplicity at glutamatergic synapses onto interneurons in stratum radiatum**. (A_1_) A typical recording at -80 mV from an interneuron in stratum radiatum from a P17 rat, upper trace shows AMPA sEPSCs under control conditions and lower trace shows mEPSCs after wash-in of TTX. (A_2_) At ages P7 - P18, TTX does not change the AMPA EPSC amplitude (an average reduction of 1.2 ± 0.8 pA, P = 0.2, n = 4, two-tailed paired t-test). (A_3_) TTX reduces AMPA EPSC frequency by 4.3 ± 1.1 Hz (P = 0.01, n = 4, one-tailed paired t-test). (B_1_) A typical recording at -80 mV from an interneuron in stratum radiatum from a P50 rat, upper trace shows AMPA sEPSCs under control conditions and lower trace shows mEPSCs after wash-in of TTX. (**B**_2_) At ages P30 - P50, TTX does not affect the AMPA EPSC amplitude (an average reduction by 0.3 ± 0.9 pA, P = 0.8, n = 6, two-tailed paired t-test). (B_3_) TTX reduces AMPA EPSC frequency by 2.4 ± 0.5 Hz (P = 0.003, n = 6, one-tailed paired t-test). (C) The average multiplicity was 1.1 ± 0.1 (P = 0.09, n = 4, one-tailed one-sample t-test with test value = 1) for the young age group (P7-18, open circles) and 1.1 ± 0.1 (P = 0.17, n = 6) for the older rats (> P30, filled circles). (D) PPR measurements from 8 interneurons in stratum radiatum (red filled circles) and 7 pyramidal cells (green filled circles). The average PPR (50 ms) for the interneurons in stratum radiatum is 1.17 + 0.19/- 0.16 (geometric mean +/- SEM) and for the pyramidal cells 1.43 + 0.22/- 0.19 (P = 0.4, two-tailed Student's t-test). Black filled circles show mean ± SEM and green and red open and filled circles show individual experiments. Asterisks denote the level of significance as follows: *: P < 0.05, **: P < 0.01.

**Figure 3:**
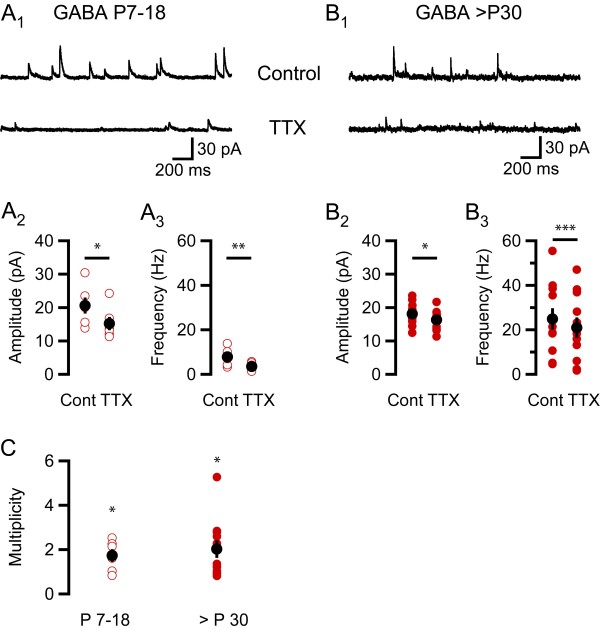
**GABA synapses onto interneurons in stratum radiatum show a multiplicity higher than one already in the developing rats**. (A_1_) A typical recording at 0 mV from an interneuron in stratum radiatum from a P9 rat, upper trace shows GABA sIPSCs under control conditions and lower trace shows mIPSCs after wash-in of TTX. (A_2_) TTX reduces the GABA IPSC amplitude by 5.4 ± 2.0 pA (P = 0.04, n = 6, two-tailed paired t-test). (A_3_) TTX reduces GABA IPSC frequency by 4.3 ± 1.0 Hz (P = 0.0045, n = 6, one-tailed paired t-test). (B_1_) A typical recording at 0 mV from an interneuron from a P43 rat, upper trace shows GABA sIPSCs under control conditions and lower trace shows mIPSCs after wash-in of TTX. (B_2_) TTX reduces the GABA IPSC amplitude by 1.8 ± 0.7 pA (P = 0.02, n = 11, two-tailed paired t-test). (B_3_) TTX reduces GABA IPSC frequency by 3.9 ± 0.7 (P < 0.001, n = 11, one-tailed paired t-test). (C) The average multiplicity was 1.7 ± 0.3 (P = 0.025, n = 6, one-tailed one-sample t-test with test value = 1) for the young age group (P7-16, open circles) and 2.0 ± 0.4 (P = 0.014, n = 11, one-tailed one-sample t-test with test value = 1) for the older rats (> P30, filled circles). Black filled circles show mean ± SEM and red open and filled circles show individual experiments. Asterisks denote the level of significance as follows: *: P < 0.05, **: P < 0.01, ***: P < 0.001.

In contrast to the gradual establishment of AMPA EPSC multiplicity onto pyramidal cells during development [4], GABAergic synapses onto pyramidal cells showed a high and developmentally stable multiplicity [5]. Consistent with that study, we found that IPSC amplitude and frequency were reduced in pyramidal cells from young adult (P30-60) animals after TTX was washed in (an average reduction of 8.4 ± 2.7 pA, P = 0.03, two-tailed paired t-test, and 3.2 ± 0.8 Hz, P = 0.005, one-tailed paired t-test, respectively, n = 6, Figure 1, 2, 3). The multiplicity was 2.4 ± 0.4 (P = 0.007, n = 6, one-tailed one-sample t-test with test value = 1, Figure 1B4), thus comparable to what has previously been reported [5].

### Connectivity at pyramidal cell - CA1 interneuron in stratum radiatum connections

**Figure 4:**
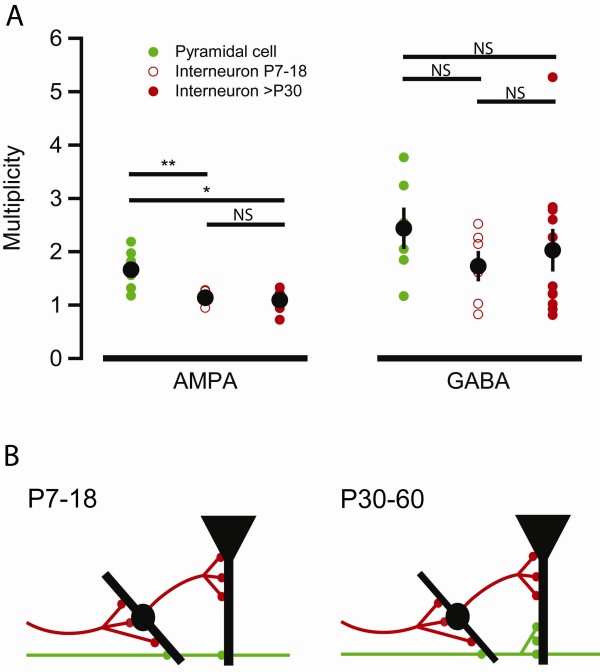
**Summary and schematic model**. (A) Summary of the multiplicity data obtained in Figures 1-3. ANOVA and Bonferroni post hoc test shows a significant difference between AMPA multiplicity in pyramidal cells from young adult animals and AMPA multiplicity in interneurons of both P7-18 rats and young adult rats (P = 0.004 and P = 0.02, respectively, left panel), but no significant differences between the groups of the right panel. (B) Schematic model illustrating the development of connectivity onto pyramidal cells and interneurons in stratum radiatum. Inhibitory (red) axons form multiple connections already in the P7-18 rats, whereas the excitatory (green) axons start out with single release site connections (left panel). During development excitatory axons grow multi release site connections onto pyramidal cells, but not to interneurons in stratum radiatum (right panel).

We next recorded AMPA sEPSCs and mEPSCs in interneurons in stratum radiatum from developing (P7-18) and young adult (P30-60) interneurons (Figures 2A, B). In the developing group the average amplitude reduction was 1.2 ± 0.8 pA (P = 0.2, n = 4, two-tailed paired t-test, Figure 2A2) and the average reduction in frequency was 4.3 ± 1.1 Hz (P = 0.01, n = 4, one-tailed paired t-test, Figure 2A3. The young adult group showed an average reduction in amplitude by 0.3 ± 0.9 pA (P = 0.8, n = 6, two-tailed paired t-test, Figure 2B2) and in frequency by 2.4 ± 0.5 Hz (P = 0.003, n = 6, one-tailed paired t-test, Figure 2B3) Interneurons are highly diverse and the large observed variance in amplitude and frequency (cf. Figures 2A, B) might in part be explained by this. Interestingly, despite this interneuron heterogeneity, the variability in multiplicity is very small in both age groups. Further, we found no significant deviation from a multiplicity of one at glutamatergic connections onto these interneurons, neither from developing nor from young adult rats (Figure 2C). The result from the older group (1.1 ± 0.1, n = 6) is thus in marked contrast to, and significantly different (P = 0.004, oneway ANOVA with Bonferroni post hoc, also including young group, cf. Figure 4A) from, the result from the pyramidal cells (1.7 ± 0.1, n = 8) of the same age. A possible explanation for this finding would be if glutamate synapses onto interneurons in stratum radiatum have a substantially lower release probability than those onto CA1 pyramidal cells. Previous studies have, however, shown that these groups of synapses, on average, exhibit similar release probabilities [16,17]. To get an estimate of the average release probability in glutamatergic synapses onto interneurons in stratum radiatum, under our conditions of high Ca^2+ ^and low Mg^2+^, we calculated paired-pulse ratios (PPRs). The average PPR was 1.2 + 0.19/- 0.16 (n = 8, geometric mean and SEM, see Methods, Figure 2D), which is comparable to that obtained from pyramidal cells under the same conditions (1.4 + 0.22/- 0.19, n = 7, Figure 2D). This similarity in PPR values (P = 0.4, two-tailed Student's t-test) indicates that the absence of multiplicity at glutamatergic connections onto these interneurons is not because of low average Pr.

### Connectivity at GABAergic interneuron - interneuron in stratum radiatum connections

Figure 3 illustrates the effect of TTX on the amplitude and frequency of GABA sIPSCs recorded in interneurons in stratum radiatum from developing (P7-18) and young adult (P30-60) rats. There was a large variability in both amplitude and frequency of GABA IPSCs, especially in the older age group (Figures 3A-B), possibly related to the presence of different types of interneurons. In recordings from developing animals the amplitude, on average, decreased by 5.4 ± 2.0 pA (P = 0.04, n = 6, two-tailed paired t-test, Figure 3A2) and the frequency by 4.3 ± 1.0 Hz (P = 0.005, one-tailed paired t-test, n = 6, Figure 3A3). Recordings from young adult animals showed an average reduction in amplitude by 1.8 ± 0.7 pA (P = 0.02, n = 11, two-tailed paired t-test, Figure 3B2) and frequency by 3.9 ± 0.7 (P < 0.001, n = 11, one-tailed paired t-test, Figure 3B3). A multiplicity higher than one was evident in both age groups (1.7 ± 0.3, P = 0.03, n = 6 and 2.0 ± 0.4, P = 0.01, n = 11, respectively, one-tailed one-sample t-test with test value = 1), but noticeably already in the developing group (Figure 3C). The pattern of GABAergic multiple connectivity onto interneurons in stratum radiatum shown here is similar to that previously found in pyramidal cells [5].

The findings from this study are summarized in Figure 4A. One-way ANOVA with the Bonferroni post hoc test shows that the multiplicity at glutamatergic synapses onto both age groups of interneurons in stratum radiatum is significantly lower than the multiplicity at glutamatergic synapses onto pyramidal cells (Figure 4A, left panel). There are, on the other hand, no significant differences between the three different groups of data from GABAergic synapses (Figure 4A, right panel).

## Discussion

In this study, using the multiplicity method [4], we have compared the development of average glutamatergic and GABAergic synaptic connectivity onto interneurons in stratum radiatum with that found previously onto CA1 pyramidal cells [4,5,15]. We found that the interneurons in stratum radiatum receive synaptic inputs with an average multiplicity higher than one from presynaptic GABAergic neurons (Figures 3, 4A). In agreement with what was previously found for the GABAergic innervation of the CA1 pyramidal cells (Figures 1B, 4A) [5], this multiple connectivity existed already from the early postnatal development. In contrast, the development of the glutamatergic connectivity at the interneurons differed markedly from that previously found at the CA1 pyramidal cells. Thus, our results suggest that an average connectivity corresponding to one functional single release site persists into adulthood onto interneurons in stratum radiatum (Figures 2, 4A), whereas it develops to multi-release site connections onto pyramidal cells [4] (Figure 1A, 4A). The conclusions from the present study, together with previous studies using the multiplicity method, are schematically summarized in Figure 4B. Collectively these studies suggest that the development of synaptic connectivity in this region follow neuron-specific rules with instructive signals from both pre- and postsynaptic elements. The importance of the presynaptic phenotype is illustrated by the difference in development of GABAergic and glutamatergic connectivity onto the same postsynaptic neurons. The importance of the postsynaptic phenotype is illustrated by the divergent development of connectivity between glutamatergic neurons and GABAergic or glutamatergic neurons, respectively. This differential control of average connectivity may be useful in future studies examining molecular mechanisms governing the degree of connectivity between neurons.

### Structural basis for multiplicity

The multiplicity approach, used in the present study to determine connectivity, has several limitations. First, it provides an average estimate and does not reveal the heterogeneity in number of connections between cell pairs. Second, we have recorded from interneurons with their somata in the stratum radiatum. This selection more likely includes CCK-positive Schaffer-collateral associated and apical dendrite innervating interneurons, than for instance parvalbumin-positive interneurons [18]. It is thus possible that there are other GABAergic interneurons with connectivity patterns that depart from that described here. Indeed, glutamatergic neurons can form multiple connections onto interneurons in CA1 strata oriens-alveus [14]. Moreover, since the population of presynaptic GABAergic neurons, in contrast to the population of presynaptic glutamatergic neurons, is diverse in for example spiking properties, certain types of GABAergic interneurons may contribute disproportionally to the average multiplicity. Third, there are reasons to assume that somatic whole cell recordings of spontaneous synaptic currents fail to detect distal, electrotonically remote, events [19,20]. Thus, results obtained by the multiplicity method may be preferentially relevant for proximal synapses. Fourth, a major unanswered question in the present study is the structural basis for the multiplicity. One can envisage three different scenarios: i/a single release site synapse that allows multivesicular release to increase transmitter concentration at the same set of postsynaptic receptors; ii/a multi release site synapse where released transmitter acts on independent sets of postsynaptic receptors; iii/several morphologically distinct single release site synapses. For GABAergic connections there is morphological evidence that a single axon can form several distributed synaptic connections onto the same postsynaptic neuron [10,11]. Distributed glutamatergic connectivity has been shown in the neocortex [21,22], and may also occur between the Schaffer collaterals and pyramidal cells [23]. Thus, morphologically distributed synapses is a possible explanation for multiple connectivity. On the other hand, there is electrophysiological evidence indicating that, at least under conditions of high release probability, GABA synapses [24] and glutamate synapses [25-27] onto mature CA1 pyramidal cells are capable of multivesicular release. In contrast to these findings in mature CA3-CA1 glutamate synapses, electrophysiological studies of developing (during the first two postnatal weeks in the rodent) CA3-CA1 synapses have shown that these connections have univesicular release [4,8,15,28]. For glutamate synapses the issue of multi- versus univesicular release is complicated by whether AMPA or NMDA receptors are used as reporters in electrophysiological studies [27]. This is because NMDA receptors have a higher affinity for glutamate, and thus are more likely to respond to intrasynaptic spillover than AMPA receptors. A possible morphological correlate for the development from single release site to multi-release site glutamate synapses is the progressive development from shaft and thin spine synapses to stubby and mushroom synapses [29]. This spine development is largely consistent with multiplicity development [4]. The absence of spines on most GABAergic interneurons in stratum radiatum of the hippocampus also supports the idea that spines in GABAergic interneurons reflect multiple glutamatergic innervation.

### Development of multi release site connections

A key difference between glutamatergic and GABAergic synaptic connections is that GABAergic connections appear to form as multiple contacts [30] (Figure 3), whereas glutamatergic connections are formed as single release site connections and may, on principal neurons, attain multiple connectivity later in development.

The single release site connectivity for glutamatergic connections in the CA1 region is largely preserved throughout development and into adulthood onto interneurons, but is progressively complemented by multi release site connections onto pyramidal cells. What governs this development towards multi release site glutamatergic connections is not known. One attractive hypothesis is that it reflects learning based synaptic strengthening, since it is generally thought that the ultimate consequence of associative long-term potentiation (LTP) is an increase in connectivity [31]. A corollary of this hypothesis is that LTP early in development, and LTP at interneuronal glutamate synapses, do not result in increased connectivity.

In fact, there are several results arguing that LTP at glutamatergic synapses onto interneurons is mechanistically distinct from that at mature glutamatergic synapses onto pyramidal cells. Two major distinct types of glutamate synapses onto interneurons have been described; those with calcium permeable AMPA receptors and few, or no, NMDA receptors and those with calcium impermeable AMPA receptors and abundant NMDA receptors [32,33]. The induction of LTP at the first type of synapse is NMDA receptor independent, but relies on metabotropic glutamate receptors (mGluRs) and calcium permeable AMPA receptors and appears to be expressed as an increase in release probability [34,35]. The induction of LTP at the second type of synapse is NMDA receptor dependent, is not associated with an accompanying change in paired-pulse ratio [36], and may well involve unsilencing of AMPA silent synapses, which are frequent at mature interneurons [33], but largely absent in mature pyramidal cells [7,37,38]. Thus, at both types of glutamatergic synapses onto interneurons, LTP appears to potentiate without increasing the number of functional release sites.

## Conclusions

Glutamate and GABA synaptic connectivity in the hippocampal CA1 region develop very differently, not only onto pyramidal cells, but also onto interneurons in stratum radiatum (Figure 4). Thus, whereas average GABA connectivity is multiple throughout the development, glutamate connectivity is unitary early in development. Our results further suggest that the development of glutamate synaptic connectivity differs markedly between pyramidal cells and GABAergic interneurons in stratum radiatum, such that a given presynaptic CA3 pyramidal cell appears not allowed to increase its connectivity onto the postsynaptic interneuron in stratum radiatum, as it may do onto the postsynaptic CA1 pyramidal cell.

## Methods

### Slice preparation and solutions

Experiments were performed on hippocampal slices from 7-18 (developing, n = 5) day old female and male or 30-60 (young adult, n = 27) day old male Wistar rats (in house breeding). The animals were killed in accordance with the guidelines of the local ethical committee for animal research. Rats were anesthetized by inhalation of isoflurane (Abbott, Abbott Park, Il, USA; 1 ml of isoflurane in a 5 l box until breathing slowed down markedly and the limb withdrawal reflex was abolished) prior to decapitation. The brain was removed and placed in an ice-cold solution containing (in mM): 140 cholineCl, 2.5 KCl, 0.5 CaCl_2_, 7 MgCl_2_, 25 NaHCO_3_, 1.25 NaH_2_PO_4_, 0.5 ascorbic acid, and 7 D-glucose. Transverse hippocampal slices (300 or 400 μm thick) were cut with a vibratome (Slicer HR 2, Sigmann Elektronik, Hueffenhardt, Germany) in the same ice-cold solution, and they were subsequently stored in a solution containing (in mM): 124 NaCl, 3 KCl, 2 CaCl_2_, 4 MgCl_2_, 26 NaHCO_3_, 1.25 NaH_2_PO_4_, 0.5 ascorbic acid, 3 myo-inositol, 4 D,L-lactic acid, and 10 D-glucose at 25°C. After ≥ 60 min of storage, a single slice was transferred to a recording chamber where it was kept submerged in a constant flow (~4.4 ml/min) at 30-32°C. The perfusion solution contained (in mM): 124 NaCl, 3 KCl, 4 CaCl_2_, 0.5 MgCl_2_, 26 NaHCO_3_, 1.25 NaH_2_PO_4_, and 10 D-glucose. D-AP5 (50 μM) was always present in the perfusion solution to block *N*-methyl-D-aspartate (NMDA) receptor mediated activity. All solutions were continuously bubbled with 95% O_2 _and 5% CO_2 _(pH ~7.4). In these experiments we used a high concentration of calcium (4 mM) and a low concentration of magnesium (0.5 mM) in the extracellular solution [cf. [4]] to ascertain a high release probability.

### Recording and analysis

Whole cell patch-clamp recordings were performed on CA1 pyramidal cells or stratum radiatum interneurons that were visually identified using infrared-differential interference contrast video microscopy (CV-M50 IR, JAI Corp., Japan) mounted on a Nikon E600FN microscope. Interneuron somata were round, oval, or fusiform and two, or more, proximal dendrites could be clearly identified. Care was taken to exclude cells with their somata in the stratum lacunosum-moleculare, and to exclude the so called radiatum giant cells [39] by avoiding cells that were pyramidal shaped and had only one prominent proximal dendrite. For whole-cell recordings the pipette solution contained (in mM): 130 Cs-methanesulfonate, 2 NaCl, 20 HEPES, 0.6 EGTA, 5 QX-314, 4 Mg-ATP, and 0.4 GTP (pH ~7.3 and osmolality 280-300 mOsm). Patch pipette resistances were 2-5 MΩ. PSCs were recorded at a sampling frequency of 10 kHz and filtered at 3 kHz, using an EPC-10 amplifier (HEKA Elektronik, Lambrecht, Germany). Cells were voltage-clamped at -80 mV for alpha-amino-3-hydroxy-5-methyl-4-isoxazoleprepionic acid (AMPA) EPSC (excitatory PSC) recordings or at 0 mV for GABA IPSC (inhibitory PSC) recordings. The liquid junction potential was both measured and calculated to be about 8 mV and was not corrected for. Series resistance was monitored using a 20 ms 10 mV hyperpolarizing pulse. The series resistance was not allowed to exceed 20 MΩ in whole-cell recordings, or to change more than 20% during an experiment, otherwise the experiment was discarded. Experiments were also discarded if the PSC frequency did not decrease at least 15% or, for recordings with sPSC frequency > 8 Hz, more than 1 Hz, after the application of tetrodotoxin (TTX, 0.1 - 0.5 μM). Spontaneous PSC analysis was based on recordings at a given membrane potential for at least 60-120 s or a minimum of 100 sPSCs. Recordings were transferred into the Mini-Analysis Program (version 6.0.7; Synaptosoft Inc., Decatur, GA, USA) and were checked in segments of ~ 400 ms. All events visually judged as PSCs were manually indicated for additional analysis in the Mini-Analysis Program. The multiplicity [4] was calculated for each individual experiment using IGOR Pro software (WaveMetrics, Lake Oswego, OR, USA). F_s _and F_m _denote the frequencies of sPSCs and mPSCs, respectively, and Q_s _and Q_m _denote the amplitudes:

$$\text{Multiplicity }=\;\left[\left({\text{F}}_{\text{s}}\times {\text{Q}}_{\text{s}}\right)\;-{\text{F}}_{\text{m}}\times {\text{Q}}_{\text{m}}\right)]/[\left({\text{F}}_{\text{s}}\;-{\text{F}}_{\text{m}}\right)\;\times {\text{Q}}_{\text{m}}]$$

Previous studies have shown that the frequency of mPSCs is stable over time in whole-cell recordings and is not affected by TTX [4,40].

For recording of evoked EPSCs, Schaffer collateral/commissural afferents were stimulated using 0.2 ms biphasic (negative/positive) constant current pulses (5-20 μA; STG 1002, Multi Channel Systems, Reutlingen, Germany) delivered through an insulated tungsten microelectrode (resistance ~ 0.3-0.5 MΩ). Stimulation electrodes were positioned in the stratum radiatum and synaptic inputs received a paired pulse stimulation (50 ms stimulus interval) every 5 s or every 1 s. Evoked responses were analyzed off-line using custom-made IGOR Pro software. AMPA EPSC amplitudes were measured on averages of 12 to 60 consecutive recordings. PPR data is presented as geometric mean, i.e. the logarithm for the PPR was used for averaging. This procedure circumvents the risk of obtaining false increases in the PPR because of averaging individual ratios [cf. [41]].

Data is expressed as mean ± SEM. Statistical significance was evaluated using PASW Statistics 18 (SPSS Inc., Chicago, IL, USA) software, using paired *t*-test or Student's *t-*test unless otherwise is indicated. One-tailed test was used for multiplicity data since it cannot take values below 1 and for frequency reductions since there has to be a reduction due to the inclusion criteria.

### Drugs

TTX, D-AP5 and QX-314 were purchased from Ascent scientific, Bristol, UK.

## Authors' contributions

IR collected and analyzed the data. EH conceived the study. EH and IR designed the study and drafted the manuscript. Both authors read and approved the manuscript in its final form.
